# An Examination of Burnout Predictors: Understanding the Influence of Job Attitudes and Environment

**DOI:** 10.3390/healthcare8040502

**Published:** 2020-11-20

**Authors:** Katelyn J. Cavanaugh, Hwa Young Lee, Diane Daum, Shine Chang, Julie G. Izzo, Alicia Kowalski, Courtney L. Holladay

**Affiliations:** 1 Leadership Institute, University of Texas MD Anderson Cancer Center, Houston, TX 77030, USA; KJCavanaugh@mdanderson.org; 2 Cancer Prevention Training Program, University of Texas MD Anderson Cancer Center, Houston, TX 77030, USA; HLee12@mdanderson.org; 3 CultureIQ, Huntersville, NC 28078, USA; diane.daum@cultureiq.com; 4 Epidemiology, University of Texas MD Anderson Cancer Center, Houston, TX 77030, USA; shinechang@mdanderson.org; 5 Human Resources Administration, University of Texas MD Anderson Cancer Center, Houston, TX 77030, USA; jizzo@mdanderson.org; 6 Anesthesiology & Peri-Operative Medicine, University of Texas MD Anderson Cancer Center, Houston, TX 77030, USA; amkowalsk@mdanderson.org

**Keywords:** burnout, job attitudes, predictors

## Abstract

Burnout amongst healthcare employees is considered an epidemic; prior research indicates a host of associated negative consequences, though more research is needed to understand the predictors of burnout across healthcare employees. All employees in a cancer-focused academic healthcare institution were invited to participate in a bi-annual online confidential employee survey. A 72% response rate yielded 9979 complete responses. Participants completed demographic items, a validated single-item measure of burnout, and items measuring eight employee job attitudes toward their jobs and organization (agility, development, alignment, leadership, trust, resources, safety, and teamwork). Department-level characteristics, turnover, and vacancy were calculated for group level analyses. A univariate F test revealed differences in burnout level by department type (*F* (3, 9827) = 54.35, *p* < 0.05) and post hoc Scheffe’s tests showed employees in clinical departments reported more burnout than other departments. Hierarchical multiple regression revealed that employee demographic and job-related variables (including department type) explained 8% of the variance of burnout (*F* (19, 7880) = 37.95, *p* < 0.001), and employee job attitudes explained an additional 27% of the variance of burnout (*F* (8, 7872) = 393.18, *p* < 0.001). Relative weights analysis at the group level showed that, of the constructs measured, alignment is the strongest predictor of burnout, followed by trust and leadership. The relationships are inverse in nature, such that more alignment is related to less burnout. Turnover and vacancy rates did not predict group level burnout. The results reported here provide evidence supporting a shift in the focus of research and practice from detection to prevention of employee burnout and from individual-focused interventions to organization-wide interventions to prevent burnout.

## 1. Introduction

Burnout is widely recognized as permeating the healthcare field, to the point that it is referred to as an epidemic [1]. Prior research has shown that a third to over half of physicians across all levels of training, specialties, and practice patterns have reported symptoms in national surveys in the USA [2]. Much of the burnout literature in healthcare organizations has focused on the negative outcomes related to burnout of specific clinical care provider groups, finding increased alcohol abuse [3], decreased job satisfaction [4,5,6], and discontent with work–life balance [7]. At the organization level, evidence suggests that burnout increases employee intentions to leave jobs or retire early [7], which is likely to lead to actual turnover [8] and decrease productivity [9,10]. These effects can impact patient care [11], negatively affecting patient communication [12,13] and satisfaction [14,15,16], and increasing medical errors [17], infection rates [18], and even patient mortality rates [19]. Conservative estimates of the total cost of burnout are $5–10 billion per year [20]. What is currently missing from broader understanding of burnout in healthcare settings is better conceptualization of the processes and mechanisms by which burnout occurs within healthcare organizations. With more information, investigations to predict and interventions to prevent burnout may be developed.

To fill this gap, it is important to recognize that healthcare institutions are comprised of multiple components and multidisciplinary teams with unique functions, such as patient care, research, administration, and training [21]. As might be expected by the attraction-selection-attrition model, individuals who choose to apply to, are selected into, and stay in such organizations are often similar to each other across roles [22] and may have similar responses to burnout; however, this is untested. The existing burnout literature in healthcare organizations has focused only on specific professional types, such as physicians or nurses, ignoring the many other roles that are vital to patient care and organizational functioning. It leads to flaws in the research design and methodology that are needed to accommodate the variety of individuals and how they work together. 

Beyond this more holistic, systems-wide approach, the current study draws from previous work in the organizational psychology research literature, which has identified the predictors of burnout across a wide range of industries, organizations, and job types [23,24]. In particular, job attitudes are multifaceted components that include cognitive and affective aspects defined as evaluative feelings, beliefs, and attachment to a job [25], such as job satisfaction, involvement, empowerment, organizational commitment, alignment, and perceived organizational support. These are known to relate to burnout [26]. However, a clearer definition of the construct of job attitudes is needed, and it is not known which components of the construct are most related to burnout. Investigating and better understanding the components of job attitudes that impact burnout in healthcare institutions will allow these organizations to design more effective interventions to prevent burnout and the accompanying negative impacts.

With this focus in mind, we first identify the construct of job attitudes using confirmatory factor analysis to determine whether the factor structure is consistent with our initial understanding of the concept. We then analyzed the two levels of data: individuals and departments. The level of burnout between individuals and departments may differ depending on department types (clinical, clinical-research, research, non-clinical/non-research) and environment (e.g., turnover rate). The research questions for the current study were: Which departments report more burnout? Which components of job attitudes are more related to burnout at the individual and department levels? Are both turnover and vacancy rates important predictors of burnout after taking into account job attitudes at the department level? We used survey methodology to investigate institution-wide predictors of burnout for all employees within a large single healthcare and research institution focused on cancer. To our knowledge, this is the first study to measure burnout across all departments and roles, and to identify both individual and organizational predictors of burnout across all employees within one healthcare organization. Our study is an important step to analyze predictors of burnout and better enable organizations to target interventions that mitigate burnout.

## 2. Method

### 2.1. Participants

All 19,846 employees and trainees of a cancer-focused academic research institution were invited to participate in a bi-annual online confidential employee survey for a two-week period in April 2017. The survey was designed to facilitate conversations between management and employees regarding issues impacting the work environment, and any barriers that employees were facing in their work. To promote candid responses about the institution, jobs, and other related issues, the online survey was hosted by an external consultant company to manage anonymity of respondents. An email invitation was sent with a survey link and respondents verified their department and positions before starting the survey. Employees received one system-generated reminder to participate, about half-way through the administration window. In addition, the institution reported the organization’s response rate daily and local leaders sent emails toward the beginning, middle, and end of the administration window to encourage participation. A 72% response rate was achieved, with 14,213 employees completing the survey. Thirty percent of participants skipped three or more demographic questions. Thus, 9979 individuals’ responses were used for analyses. Participants self-reported demographics, including gender, supervisory role, employee type, mentoring participation, length of service, race, and generation. Demographics by department type are shown in Table 1. IRB approval was received for the analysis of these data (IRB Protocol number # PA19-0314). The authors received no financial support for the research, authorship, and/or publication of this article.

### 2.2. Measures

**Burnout.** Burnout was measured with a single item from the Physician Worklife Study: “Overall, based on your definition of burnout, how would you rate your level of burnout?” [27,28]. The full Maslach Burnout Inventory (MBI) could not be used due to concerns about overall survey length (as employee job attitudes were also being measured). Scores range from 1–5 and higher scores indicate a more favorable response (less burnout). Concurrent validity research shows that this single item is comparable to the emotional exhaustion construct of burnout as measured by the 22-item version of MBI [29,30] and the item is widely used in health professional research to effectively measure burnout [31]. The full item can be found in Appendix A.

**Employee Job Attitudes.** Employee job attitude scales were designed to measure eight constructs of employees’ attitudes towards their jobs and organization on a 5-point Likert-type scale. The eight constructs were agility, development, alignment, leadership, trust (measuring respect, trust, inclusion, and diversity), resources, safety, and teamwork. Some items used were written specifically for this survey, others were provided by an external consulting company for benchmarking purposes. Scales were not intended to be comprehensive measures of each construct, but rather to pinpoint specific concerns of management based on the strategic direction of the organization and on past employee feedback. Means and standard deviations (SD), Cronbach’s alphas, and sample items which are exemplars from each construct are shown in Appendix B. Confirmatory factor analysis was conducted to confirm the factor structure of employee job attitudes, and results showed adequate fit [32,33,34] (*χ*^2^ (296) = 5614.58, Root Mean Square Error of Approximation (RMSEA) = 0.04 [90% CI = 0.41, 0.43], Comparative Fit Index (CFI) = 0.94, Tucker-Lewis Index (TLI) = 0.93, Standardized Root Mean Residual (SRMR) = 0.04). Correlations between burnout and each employee attitude construct are shown in Table 2.

For the computation of job turnover rates and job vacancy rates, headcount files, files of employees who terminated (both voluntarily and involuntarily), and vacancies by department were retrieved from the institution’s human resources information system for each of the seven months prior to the collection of the employee survey data. From this information, the job turnover rates and job vacancy rates were computed as described below.

**Job Turnover Rates.** Job turnover rates were computed at the department level. Counts of voluntary and involuntary departures for each department in the seven months preceding the survey were divided by department headcounts for those same months, and then annualized to create turnover rates. The voluntary and involuntary turnover variables were used as group-level predictors of burnout. 

**Job Vacancy Rates.** Vacancies were counted for each of the seven months prior to the survey and were divided by the total headcounts for those same months and averaged across the seven months. The vacancy variable was also used as a group-level predictor of burnout. 

### 2.3. Statistical Analysis

Statistical analyses were conducted to identify factors predicting burnout at both individual and group levels. At the individual level, an analysis of variance was conducted to compare the level of burnout among four department types: clinical (275 departments focused on direct patient care activities, e.g., ambulatory centers, nursing units, and providers), clinical-research (54 departments focused on translational research and bringing the research to the patient), non-clinical/non- research (119 departments focused on administrative and business functions, e.g., finance and human resources), and research (12 departments focused on laboratory and bench research). A hierarchical multiple regression analysis was then conducted to identify which variables significantly predict the level of burnout by entering an individuals’ socio-demographic and job-related characteristics including dummy coded versions of gender, race, generation, length of service, department type, mentoring relationship, supervisory status, and shift, as the first step in the model. The eight employee attitude predictors (agility, development, alignment, leadership, trust, resources, safety, and teamwork) were entered as a second step in the model, to determine whether employee job attitudes accounted for significant additional variance beyond the demographics and job characteristics. 

For a group-level analysis, employee attitude variables were aggregated to the department level. To maintain confidentiality, only responses from work units of five or more individuals were used in analysis. Among 569 total departments, 460 had five or more employee survey responses and were used for group level analysis. For some analyses, we grouped departments by functional department type. Means and standard deviations (SD) for burnout and each employee attitude construct by department can be found in Table 3.

To identify the relative importance of organization predictors on group-level burnout, a relative weight analysis using a bootstrapping technique [35,36] was conducted with multiple job attitude predictors, employee turnover rates, and vacancy rates as a group level analysis. Relative weight analysis is useful to identify the contributions of each predictor in a model. As a relative weight, the Epsilon value of each predictor was calculated to indicate the importance of each predictor in the model, taking into account both unique variance, and variance shared with other predictors. This is particularly useful for practitioners making decisions about where to focus organizational development efforts, as the interpretation is more straightforward than that of a regression coefficient. The sum of the predictors’ epsilon values yields the model’s R squared. The Epsilon values were tested for statistical significance at *p* < 0.05. 

## 3. Results

**Burnout by department type.** The univariate F test on the level of burnout yielded significant differences across four types of departments (*F* (3, 9827) = 54.35, *p* < 0.05). A post hoc Scheffe test, the most conservative multiple comparison test, showed that employees in clinical settings experienced more burnout than those in research, clinical-research, and non-clinical/non-research settings. No other comparisons were statistically significant. The Scheffe test results are shown in Table 4.

**Incremental prediction of employee job attitudes to explain burnout.** Demographic and job-related variables entered into the first step explained 8% of the variance of burnout (*R*^2^ = 0.08, *F* (19, 7880) = 37.95, *p* < 0.001). All demographic and job-related variables, except for shift, were significant predictors of burnout. Specifically, women, Caucasians, millennials, clinical department employees, and employees without a mentoring relationship had higher burnout levels (e.g., lower scores on the burnout item). In addition, individuals who worked more than 1 year compared with those with less than one year of service had significantly higher burnout level (as evidenced by lower scores on the burnout item), as did individuals in supervisory roles compared to non-supervisors (*p* < 0.05 for each). Adding eight job attitude variables to the model at the second step significantly increased the explanation of variance of burnout by 0.27 to *R*^2^ = 0.35 (*F* (8, 7872) = 393.18, *p* < 0.001). Thus, 27% additional variance in burnout was accounted for by attitudes over and above the explanation through demographics. Specifically, leadership, trust, resources, agility, and alignment were significant predictors of burnout level. Results of the hierarchical regression are shown in Table 5. 

**Aggregation of employee job attitudes and burnout at the group level.** To determine whether group level effects would be expected, ICC (1) values were computed and are shown in Table 6 [37]. ICC(1) can be considered a measure of effect size of group membership, and these were quite low, suggesting that group level data would not add any predictive information to the individual level data (e.g., the variance predicted by group membership was near 0). However, given that some of the variables of interest (Job Turnover Rate, Job Vacancy Rate) were only available at the group level, we combined them into groups to allow for the relative weights analysis and test of these predictors.

**Prediction of burnout at the group level.** The analysis showed that the model including all eight attitude predictors, the turnover rates, and vacancy rates explained 52% of the variance in burnout. All employee attitude predictors were significant predictors of burnout at the group level. In particular, the epsilon value of the alignment predictor was twice as large as other predictors. Two predictors, trust and leadership, each contributed approximately 8% of R-squared in the model. The predictor resources also accounted for 7% of variance in group level burnout. Turnover rates and vacancy rates, however, were not significant predictors of burnout. The Epsilon value of each predictor is shown in Table 7.

## 4. Discussion

Overall, results indicate that employees in clinical departments reported more burnout than those in other types of departments, and that demographic characteristics had a comparatively small impact on burnout; employee job attitudes predominately drove burnout. Of eight employee attitude constructs, the variance accounted for by alignment was nearly twice that for either trust or leadership, closely followed by resources. Employee attitudes toward jobs and the organization were stronger predictors of group level burnout than even objective indicators of the work environment i.e., turnover and vacancy rates. Together, these results have important implications for future evaluation of burnout and potential interventions to prevent the issue within healthcare and research settings.

Although this is the first peer-reviewed investigation of its kind, the results reflect mechanisms already described in existing literature. High risk and fast pace alone do not cause burnout, or virtually all employees in a healthcare institution would report burnout. The attraction-selection-attrition model is widely supported in explaining that organizations are comprised of similar individuals who thrive there, because those are the individuals who choose to apply to, are selected into, and stay in such organizations [22]. Thus, healthcare organizations are likely already comprised of the individuals who are most likely to succeed within the demands of this work environment. Instead, specific areas of misfit between employees and their work environments are well-supported correlations with burnout; such misfit includes employee perceptions of a lack of control, an absence of fairness, and breakdown in community such as isolation or social conflict [38], and are well-captured by the organizational factors in this study. In a 2018 Gallup poll, over 7500 employees identified the top five factors correlating to burnout as attributed to leadership: unfair treatment at work, unmanageable workload, lack of role clarity, lack of communication and support, and unreasonable time pressure [39]. Taken together, the focus of future burnout research should shift to include organizational factors of alignment, trust, and leadership, as these capture the important influence of environment and how individuals interact with their environment to avoid burnout. 

Implications and practical recommendations for healthcare organizations are recommended in two categories. First, the drivers of burnout at a large healthcare and research institution can be identified, and with effort, could be influenced to prevent burnout [40], which has important advantages addressing over burnout at later stages [41]. Second, interventions created to prevent employee burnout should target organizational issues, because perceptions of the work environment impact burnout more than individual demographic differences amongst employees, including department type, or even objective indicators of the work environment, including turnover and vacancy rates. The results reported here show that alignment, trust, and leadership are key drivers of burnout, with alignment contributing more influence with regard to burnout, nearly twice that of the next leading constructs. It is important to emphasize that the constructs exert this impact in an inverse relationship with burnout, recognizing that improvement in any one of these likely results in the mitigation of burnout.

Thus, organization-wide efforts to improve alignment and to increase trust and build confidence in strong leadership may reduce the likelihood of burnout, and likely will have additional benefits to employees and organizations as well. In support of the influence of alignment, Maslach and Leiter highlight how a mismatch in alignment for the individual with the organization can lead to burnout [42]. Although employees in clinical departments reported more burnout, burnout was reported in other areas, and the wider reach of organization-wide interventions will have impact upon those who do not yet experience high levels. Further, interventions applied equally rather than resources and effort focused in certain areas can bolster perceptions of organizational justice [43]. Our evidence supports organizations having obstacles common throughout the institution, with local units having unique hurdles. Addressing challenges successfully at both levels is needed for improvement. Shanafelt et al. identify nine organizational strategies, such as cultivating community at work, providing resources to promote resilience and self-care, and tapping into leadership [44]. Though the domains of alignment, trust, and leadership may be fruitful places to start, rigorous evaluation of any intervention is important to determine its efficacy.

Two examples of such interventions that (a) target organizational issues such as alignment, trust, and leadership and (b) focus on preventing burnout include investing in leadership development and establishing multidisciplinary task forces. First, time and resources spent on developing employees into more effective leaders may decrease burnout across an organization, as more effective leaders in healthcare organizations have fewer direct reports reporting burnout [45,46]. Second, institution-wide task forces on professional wellness can monitor burnout and identify, establish, and promote best practices for culture and values, education and training, leadership and community building, and efficient processes.

Shanafelt et al. leverage the business case for investing in professional wellness and outline the behaviors of organizations grouped into stages [47]. The stages reflect ascending levels of organizational commitment to improving and achieving physician well-being, and together, they serve as a reference map to attaining the highest level of investment in the community’s professional wellness across an organization. On an individual level, mentorship appears to be an effective intervention, not only in this study, but throughout the literature [48,49].

The current study was representative of a large healthcare organization focused on cancer, and included both faculty and staff, working in clinical, research, and administrative departments, at a single point in time. Limitations of this study include use of a single validated item to measure burnout rather than the full 22-item Maslach Burnout Inventory, which, while seen as the gold standard, was considered too long for inclusion in the employee survey. The single item focused on the singular concept of emotional exhaustion within burnout, without examining the constructs of cynicism and lack of accomplishment. Future research could build upon this study by examining the predictors of these additional burnout dimensions. Furthermore, inclusion of items to assess the influence of personality would have been illuminating but were not appropriate for the purpose of the employee survey. Another limitation is that the results presented here may be unique to this particular institution at one point in time. Though given that our findings align with well-supported mechanisms explaining burnout (e.g., person-job misfit), they are likely to apply to other institutions. This study broadly investigated burnout rates; future research examining burnout in subgroups by population (e.g., by demographic factors and organizational rank) and over time is needed to better understand burnout within healthcare organizations. Future research is also needed to evaluate the impact of organization-wide initiatives and unit-specific efforts to prevent, detect, and address the impact of burnout on employee, patient, and organization outcomes. An additional limitation is that the CFI and TLI values in the confirmatory factor analysis were close to but did not meet the suggested thresholds. However, this is not unexpected with data that is not truly continuous [50].

The study described here is the first peer-reviewed investigation of attitudes and employee factors to predict burnout across roles and departments within one large and diverse healthcare institution. In summary, our findings show that employees in clinical departments report higher burnout levels, though organizational factors are much stronger predictors of burnout than individual factors across healthcare institution employees. This broad investigation adds importantly to the research literature of burnout as it more closely aligns with the reliance of healthcare organizations on interdisciplinary teams, not single professional roles. Given these findings, organizations should focus efforts and resources on organization-wide, evidence-based initiatives to prevent burnout. The prevention of burnout must be considered, measured, and addressed in all areas of healthcare institutions in order to meet the needs of patients in the coming years.

## Figures and Tables

**Table 1:** Demographic characteristics by department type.

	Total Sample (*n* = 9979) *n* (%)	Clinical (*n* = 5443) *n* (%)	Clinical-Research (*n* = 1166) *n* (%)	Research (*n* = 780) *n* (%)	Non-Clinical/Non-Research (*n* = 2590) *n* (%)
Gender					
Female	6461 (64.7)	3942 (72.4)	695 (59.6)	417 (53.5)	1407 (54.3)
Male	3234 (32.4)	1326 (24.4)	450 (38.6)	349 (44.7)	1109 (42.8)
NA or Prefer Not to Answer	284 (2.8)	175 (3.2)	21 (1.8)	14 (1.8)	74 (2.9)
Supervisor					
Yes	2633 (26.4)	1301 (23.9)	361 (31.0)	234 (30.0)	737 (28.5)
No	7068 (70.8)	3969 (72.9)	762 (65.4)	519 (66.5)	1818 (70.2)
NA or Prefer Not to Answer	278 (2.8)	173 (3.2)	43 (3.7)	27 (3.5)	35 (1.4)
Employee Type					
Classified Employee	6796 (68.1)	3896 (71.5)	1647 (55.4)	385 (49.3)	1868 (72.1)
Trainee	599 (5.9)	185 (3.3)	210 (18.0)	169 (21.7)	35 (1.3)
Executive	190 (1.9)	62 (1.1)	5 (0.4)	9 (1.2)	114 (4.4)
Faculty	1116 (11.2)	679 (12.5)	217 (18.6)	153 (19.6)	67 (2.6)
NA or Prefer Not to Answer	1278 (12.8)	621 (11.4)	87 (7.5)	64 (8.2)	506 (19.6)
Participating in Mentoring					
Yes	2979 (29.9)	1616 (29.7)	467 (40.1)	295 (37.8)	601 (23.2)
No	6717 (67.3)	3665 (67.3)	663 (56.9)	454 (58.2)	1935 (74.7)
NA or Prefer Not to Answer	283 (2.8)	162 (3.0)	36 (3.1)	31 (4.0)	54 (2.1)
Length of Service					
Less than 1 year	709 (7.1)	360 (6.6)	151 (13)	104 (13.3)	94 (3.6)
1 to 2 years	1497 (15)	851 (15.6)	210 (18)	136 (17.4)	300 (11.6)
3 to 5 years	2056 (20.6)	1165 (21.4)	262 (22.5)	159 (20.4)	470 (18.1)
6 to 10 years	2282 (22.9)	1254 (23)	234 (20.1)	151 (19.4)	643 (24.8)
11 to 15 years	1630 (16.3)	856 (15.7)	149 (12.8)	86 (11)	539 (20.8)
16 to 20 years	947 (9.5)	515 (9.5)	69 (5.9)	57 (7.3)	306 (11.8)
More than 20 years	684 (6.9)	343 (6.3)	72 (6.2)	72 (9.2)	197 (7.6)
NA Or Prefer Not to Answer	174 (1.7)	99 (1.8)	19 (1.6)	15 (1.9)	41 (1.6)
Race					
White	3171 (31.8)	1611 (29.6)	318 (27.3)	265 (34)	977 (37.7)
Hispanic, Latino or Spanish origin	1170 (11.7)	644 (11.8)	126 (10.8)	39 (5)	361 (13.9)
Black or African-American	1679 (16.8)	978 (18)	84 (7.2)	62 (7.9)	555 (21.4)
Asian	2195 (22)	1132 (20.8)	467 (40.1)	313 (40.1)	283 (10.9)
Other/NA/Prefer Not to Answer	1764 (17.7)	1078 (19.8)	171 (14.7)	101 (12.9)	414 (16)
Generation					
Millennial (1981–2000)	2994 (30)	1766 (32.4)	454 (38.9)	299 (38.3)	475 (18.3)
Generation X (1966–1980)	3944 (39.5)	2132 (39.2)	406 (34.8)	263 (33.7)	1143 (44.1)
Boomer/Traditionalist (1922–1965)	2461 (24.7)	1197 (22)	224 (19.2)	173 (22.2)	867 (33.5)
Other/NA/Prefer Not to Answer	580 (5.8)	348 (6.4)	82 (7)	45 (5.8)	105 (4.1)

Note: Clinical focused on direct patient care activities, e.g., ambulatory centers, nursing units, and providers), clinical-research focused on translational research and bringing the research to the patient, research focused on laboratory and bench research and non-clinical/non-research focused on administrative and business functions, e.g., finance and human resources.

**Table 2:** Correlations between employee attitude predictors and burnout.

	1	2	3	4	5	6	7	8	9
1 Agility	-								
2 Development	0.53 **	-							
3 Alignment	0.58 **	0.63 **	-						
4 Leadership	0.55 **	0.62 **	0.66 **	-					
5 Trust	0.61 **	0.63 **	0.70 **	0.66 **	-				
6 Resources	0.49 **	0.52 **	0.60 **	0.55 **	0.60 **	-			
7 Safety	0.49 **	0.49 **	0.57 **	0.58 **	0.63 **	0.59 **	-		
8 Teamwork	0.63 **	0.57 **	0.55 **	0.55 **	0.63 **	0.45 **	0.49 **	-	
9 Burnout (High Score = Less Burnout)	0.38 **	0.39 **	0.53 **	0.44 **	0.45 **	0.39 **	0.37 **	0.35 **	-

Note. Positive values indicate positive relationships with a more favorable response (less burnout). N = 9979. ** *p* < 0.01.

**Table 3:** Mean (SD) of employee attitude predictors and burnout by department type.

	Total (9979)		Clinical (5443)		Clinical-Research (1166)		Research (780)		Non-Clinical/Non-Research (2590)	
	M	SD	M	SD	M	SD	M	SD	M	SD
Agility	3.91	0.89	3.84	0.92	4.08	0.75	4.05	0.76	3.94	0.90
Development	3.99	0.64	3.95	0.67	4.06	0.58	4.00	0.59	4.03	0.63
Alignment	3.75	0.92	3.70	0.94	3.95	0.84	3.93	0.83	3.69	0.91
Leadership	3.88	0.90	3.82	0.94	4.01	0.83	3.98	0.81	3.92	0.87
Trust	3.90	0.71	3.85	0.75	4.04	0.62	4.03	0.63	3.91	0.69
Resources	3.94	0.72	3.88	0.75	4.05	0.66	4.02	0.66	4.00	0.69
Safety	3.51	0.88	3.42	0.91	3.67	0.80	3.69	0.78	3.58	0.86
Teamwork	4.04	0.75	3.98	0.77	4.18	0.66	4.16	0.65	4.07	0.76
Burnout (High Score = Less Burnout)	3.85	1.01	3.73	1.04	4.04	0.97	4.02	0.95	3.95	0.97

**Table 4:** Comparisons of mean differences in burnout (reverse coded) by department type.

Comparison (Mean)	Estimated Mean Difference	Standard Error of Difference	Scheffe 95% CI
Clinical (3.73) vs. Clinical-Research (4.04)	0.31 *	0.03	−0.40, −0.22
Clinical (3.73) vs. Non-Clinical/Non-Research (3.95)	−0.22 *	0.02	−0.28, −0.15
Clinical (3.73) vs. Research (4.02)	−0.29 *	0.04	−0.40, 0.18

Notes. Estimated means appear in parentheses. Scores range from 1 to 5, and higher mean scores indicate a more favorable response (less burnout). * *p* < 0.05.

**Table 5:** Hierarchical multiple regression for demographic and attitude predictors of individual level burnout.

	Model 1	Model 2
Variable	*B* [95% CI]	*b* [95% CI]
Demographic and Job-Related Variables		
Gender	0.06 ** [0.04, 0.08]	0.06 ** [0.05, 0.08]
Ethnicity (Hispanic)	0.11 ** [0.08, 0.13]	0.07 ** [0.05, 0.09]
Ethnicity (African American)	0.11 ** [0.09, 0.14]	0.10 ** [0.08, 0.12]
Ethnicity (Asian)	0.19 ** [0.16, 0.21]	0.10 ** [0.08, 0.13]
Generation (Gen X)	0.12 ** [0.10, 0.15]	0.09 ** [0.07, 0.11]
Generation (Boomer)	0.21 ** [0.18, 0.24]	0.14 ** [0.12, 0.17]
Length of Service (1–2 years)	−0.10 ** [−0.14, −0.07]	−0.05 ** [−0.08, −0.02]
Length of Service (3–5 years)	−0.16 ** [−0.20, −0.12]	−0.07 ** [−0.10, −0.04]
Length of Service (6–10 years)	−0.22 ** [−0.25, −0.17]	−0.12 ** [−0.15, −0.09]
Length of Service (11–15 years)	−0.21 ** [−0.24, −0.17]	−0.12 ** [−0.15, −0.09]
Length of Service (16–20 years)	−0.15 ** [−0.18, −0.12]	−0.10 ** [−0.13, −0.07]
Length of Service (>20 years)	−0.14 ** [−0.17, −0.11]	−0.10 ** [−0.12, −0.07]
Department (Clinical-Research)	0.07 ** [0.05, 0.09]	0.04 ** [0.02, 0.06]
Department (Non-Clinical/Non-Research)	0.09 ** [0.07, 0.11]	0.04 ** [0.02, 0.06]
Department (Research)	0.05 ** [0.03, 0.08]	0.03 ** [0.01, 0.05]
Mentoring Participation	0.04 ** [0.02, 0.06]	−0.02 * [−0.04, 0.00]
Supervisory Role	−0.04 ** [−0.07, −0.02]	−0.07 ** [−0.09, −0.05]
Shift (Evening)	−0.02 [−0.04, 0.00]	0.00 [−0.02, 0.02]
Shift (Night)	−0.01 [−0.03, 0.01]	−0.01 [−0.03, 0.01]
Attitude Variables		
Teamwork		0.02 [0.00, 0.05]
Development		0.02 [0.00, 0.05]
Leadership		0.06 ** [0.03, 0.09]
Trust		0.07 ** [0.04, 0.10]
Resources		0.05 ** [0.02, 0.07]
Safety		0.01 [−0.01, 0.04]
Agility		0.03 ** [0.01, 0.06]
Alignment		0.34 ** [0.31, 0.37]
*R* ^2^	0.08	0.35
Δ *R*^2^	--	0.27 *

Note. Reference groups for the dummy coded predictors in step 1 are: Females (Gender), Caucasian (Ethnicity), Millennials (Generation), Less than 1 year (Length of Service), Clinical (Department), Not Mentored (Mentoring Participation), Non-Supervisors (Supervisory Role), Day Shift (Shift) * p < 0.05, ** *p* < 0.01.

**Table 6:** ICC (1) values for employee job attitudes and burnout.

Variable	ICC (1)
Agility	<0.01
Alignment	<0.01
Development	<0.01
Leadership	<0.01
Resources	<0.01
Trust	<0.01
Safety	<0.01
Teamwork	0.01
Burnout	<0.01

N = 460 Departments.

**Table 7:** Relative weights analysis predicting group level burnout.

Variable	Epsilon
Alignment	0.146 *
Trust	0.078 *
Leadership	0.077 *
Resources	0.069 *
Safety	0.044 *
Agility	0.042 *
Development	0.034 *
Teamwork	0.027 *
Voluntary Turnover	0.005
Involuntary Turnover	0.001
Vacancy Rate	<0.001
Total Variance accounted for	52.35%

Note. N = 460 Groups * *p* < 0.05. Higher scores on the burnout measure are indicative of lower levels of burnout.

## Appendix A. Burnout (Higher Score Is Indicative of Less Burnout)

Classify your level of burnout, using your own definition of burnout, by choosing one of the following responses:

1. I feel completely burned out and often wonder if I can go on. I am at the point where I may need some changes or may need to seek some sort of help.
2. The symptoms of burnout that I’m experiencing won’t go away. I think about frustrations at work a lot.
3. I am definitely burning out and have one or two symptoms of burnout, such as physical and emotional exhaustion.
4. Occasionally I am under stress, and I don’t always have as much energy as I once did, but I don’t feel burned out.
5. I enjoy my work. I have no symptoms of burnout.

## Appendix B

**Table A1:** Employee Job Attitudes Scales.

Scale	# Items	Sample Item	α	M	SD
Agility	3	In my work unit, we are able to implement changes quickly.	0.82	3.91	0.89
Development	3	<My organization> provides me with opportunities to learn new skills and develop myself.	0.84	3.76	0.94
Alignment	5	I can see a clear link between my work and the institution’s goals and objectives.	0.87	3.86	0.82
Leadership	3	Senior leaders inspire high performance through their leadership.	0.70	3.55	0.90
Trust	5	At <my organization> people treat one another with trust and mutual respect.	0.84	3.84	0.80
Resources	2	I have the resources I need to successfully perform all of the things I have to do at work.	0.62	4.00	0.80
Safety	2	I feel free to stop my work if I believe conditions are unsafe.	0.73	4.07	0.79
Teamwork	4	I see collaboration across different departments and groups.	0.79	4.04	0.75

Notes. N size for individual analyses was 9979 after removing individuals who skipped three or more demographics. The items were rated on a Likert-type scale ranging from 1 (*Strongly Disagree*) to 5 (*Strongly Agree*), with higher scores reflecting employees’ positive attitude scores.
